# Determining Food Insecurity: An Application of the Rasch Model with Household Survey Data in Uganda

**DOI:** 10.1155/2014/121269

**Published:** 2014-12-28

**Authors:** Abraham Owino, Ronald Wesonga, Fabian Nabugoomu

**Affiliations:** ^1^School of Statistics and Planning, Makerere University, P.O. Box 7062, Kampala, Uganda; ^2^Kyambogo University, Kampala, Uganda

## Abstract

The inexplicable nature of food insecurity in parts of Uganda and worldwide necessitated an investigation into the nature, extent, and differentials of household food security. The main objective of this study was to examine the food security dynamics and model household food insecurity. The Rasch modelling approach was employed on a dataset from a sample of 1175 (Tororo = 577; Busia = 598) randomly selected households in the year 2010. All households provided responses to the food security questions and none was omitted from the analysis. At 5 percent level of significance the analysis indicated that Tororo district average food security assessment (0.137 ± 0.181) was lower than that for Busia district (0.768 ± 0.177). All the mean square fit statistics were in the range of 0.5 to 1.5, and none of them showed any signs of distortion, degradation, or less productivity for measurement. This confirmed that items used in this study were very productive for measurement of food security in the study area. The study recommends further analysis where item responses are ordered polytomous rather than the dichotomous item response functions used. Furthermore, consideration should be given to fit models that allow for different latent distributions for households with children and those without children and possibly other subgroups of respondents.

## 1. Introduction

Food security at any level is defined as physical and economic access by all people at all times to enough, safe, and nutritious basic food to meet their dietary needs and food preferences for an active and healthy life [1–3]. Food security entails food availability, food access, and food utilization as well as stability. The International Fund for Agricultural Development concisely defined household food security as “*the capacity of a household to procure a stable and sustain-able basket of adequate food*”; adequacy may be defined in terms of quality and quantity of food, which contribute to a diet that meets the nutritional needs of all household members. Stability refers to the household's ability to procure food across seasons and transitory shortages or the long-term ability to maintain consumption levels. Sustainability covers resource use and management, human dignity, and self-reliance, among others [4]. The United Nations [1, 2] considered food and nutrition security as a key indicator of absolute poverty and physical wellbeing. Food insecurity on the other hand is a situation in which individuals do not have physical or economic access to the nourishment they need and they also have no access to resources to produce food or cash. A household is also considered food insecure if its dietary intake is less than 80% of the daily minimum recommended allowance (MRA) of caloric intake required for an individual to be active and healthy. Food insecurity may result in hunger which is a consequence of recurrent and involuntary lack of access to food. It is a severe stage of food insecurity whose measurement captures the severity of deprivation due to resource or other constraints. This situation if prolonged results in malnutrition.

Household food security is the application of the food security definition to the family level with individuals within households as the focus of concern [5] as quoted from [6].

According to [7], in addition to core characteristics of food insecurity, the other manifestations which characterize the experience of food insecurity are related household actions and reactions which are considered a first level of consequences of food insecurity. These consequences at the household level often interact with the larger environment to which the household belongs resulting in “social implications.” According to [8] a paediatric psychologist in USA, food insecurity is adversely associated with both current and future health and wellbeing of children. Quoting [9, 10], she noted that household food insecurity has insidious effects on the health and development of young children, including increased hospitalizations, poor health, iron deficiency, developmental risk and behaviour problems, primarily aggression, anxiety, depression, and attention deficit disorder. Investing in children's health and wellbeing early in life sets them on a positive trajectory toward future success.

The view of [4] is that the central conditions for bringing about improved household food security and nutritional wellbeing in a holistic and integrated manner would involve (i) a more gender sensitive participatory analysis and evaluation of project interventions from an HFS and nutrition perspective and more women-targeted interventions; (ii) the integration of health and sanitation activities and analyses through interagency collaboration; and (iii) a supportive, enabling socioeconomic, institutional and policy environment. They observe that food insecurity has detrimental linkages to disease, poor sanitation, and inadequate education that need to be addressed.

Food insecurity exists whenever food security is limited or uncertain. The measurement of food insecurity at the household or individual level involves the measurement of those quantitative, qualitative, psychological, and social or normative constructs that are central to the experience of food insecurity, qualified by their involuntariness and periodicity [11].

Measurement of household food insecurity like poverty levels in developing countries is still nondeterministic in a sense that no standard method is known to apply under all circumstances. There are many challenges involved in ascertaining the actual levels of food insecurity in an area to such an extent that death and starvation, in some places, are used as an indicator for food insecurity [12, 13]. Therefore, the basis of government and donor intervention has in most cases been not only whether the population is experiencing food insecurity, but rather the severity of food insecurity. Furness et al. [14], Anema et al. [15], and Mohammadzadeh et al. [16] have developed different measurement levels, most of them without a clear basis for categorisation other than the intrinsic meaning of the values as generated from their studies. For example, the following food insecurity measurement levels have often been used: food secure, food insecure, food insecure without hunger, and food insecure with hunger [17, 18]. Other studies add the element of children to generate categories such as food insecure without hunger with children and food insecure with hunger with children to emphasise the issue of the severity of food insecurity.

The ability to accurately measure the extent or magnitude and severity of food insecurity makes it possible to come up with more realistic, adequate, and robust ways of solving the problem [19, 20].

The main objective of this study was to examine the dynamics of food security using the Rasch modelling approach. Specifically, the study aimed at achieving the following objectives: to measure food security using the Rasch models for two contiguous districts, using consistent conventions based on statistical scaling, to estimate the stability of parameters for the estimated models, and to examine the estimated order of severity of the different measurement items.

## 2. Methodology and Data Source

Data were provided by 1,175 randomly selected households from Tororo and Busia districts in the eastern part of the country during the year 2010 using an adapted and translated questionnaire. All households provided responses to the food security questions and none was omitted from the analysis. Item-specific missing data were rare.

Table 1 gives a description of the eighteen questions that were used to capture data on the 18 indicators used for the study which were adapted from the United States Department of Agriculture set of food insecurity questions [21]. Reference period for all the 18 questions was the last 12 months prior to the day of interview. Eight of the questions focused on adults and the other ten were similar but focused on children below 18 years. For questions 1 to 6 about the food situation, the respondent was to indicate whether the statement was often true, sometimes true, or never true for a given household in the last 12 months.

Questions 7 to 13 were on coping strategies in case of food insecurity for adults and questions 14 to 18 were the same as questions 7 to 13 on coping strategies but this time they focused on children. With the exception of questions 8, 13, and 16 that were follow-up questions asking frequency of occurrence, responses to questions 7 to 18 were yes, no, or do not know.

Responses were coded into binary following standard methods so far used in the literature. For the often/sometimes/never responses, “often” or “sometimes” were coded as affirmative (value = 1), and “never” was coded as a negative response (value = 0). For yes/no responses, “yes” was coded as 1 and “no” as 0. For “how often?” responses, “almost every month” and “some months” were coded as 1 and “only 1 or 2 months” was coded as 0.

Thus, the data structure looked like Table 2.

Using [22, 23] notations and given the fact stated by the Rasch model that the log odds of a household (*v*) responding to an item (*i*) correctly are a function of ability (*θ*_*v*_) and the item's difficulty (*β*_*i*_), we state the model as in (1). Difficult items are hard to get right even for people with high ability. The odds of getting an item right decrease with item difficulty and thus the minus sign before *β*_*i*_,

(1) $$\begin{array}{c} \text{logit}\left(P_{i,v}\right)=\log\left(\frac{\mathrm{Pr}\left(P_{i,v}\right)}{1-\mathrm{Pr}\left(P_{i,v}\right)}\right)=\theta_{v}-\beta_{i}, \end{array}$$

where *v* = 1,2,…, number of households/respondents, *i* = 1,2,…, number of items, and *θ*_*v*_ is normally distributed random variable with zero mean and variance *τ*.

The Rasch model [24] programmed in *R* statistical package was used to fit, test, and generate results. It employed the item response theory whereby the probability of a household's certain reaction to a stimulus could be described as a function characterising the household's food insecurity level on a latent trait. Thus, the Rasch model [25, 26] is described as

(2) $$\begin{array}{c} P\left(X_{vi}=\frac{1}{\theta_{v}},\beta_{i}\right)=\frac{\exp\left(\theta_{v}-\beta_{i}\right)}{1+\exp\left(\theta_{v}-\beta_{i}\right)}, \end{array}$$

where *v*: 1,2,…, *n* are the households (*n*_Tororo_ = 577, *n*_Busia_ = 598), *i*: 1,2,…, *m* (*m* = 18) are the items, *X*_*vi*_: household (*v*) gives correct response to item (*i*), *θ*_*v*_ is the ability of household (*v*) to give correct response to item (*i*), and *β*_*i*_ is the difficulty level of item (*i*).

The underlying theory of the model is that if the wording of an item does not change, its estimated level of severity should not change over time. Accordingly, even if food insecurity became prevalent over time, a household at a given level of food insecurity this year is expected to respond to each item the same way a household at that level of insecurity did a year earlier.

Due to sampling variability and other factors, such as minor wording changes, we do not expect estimated model parameters to remain exactly the same over time, but a finding of significant major changes over time would call into question the model validity. Particularly problematic would be a finding of important changes in the ordering of the items by severity of food insecurity.

## 3. Findings of the Study

The Rasch model analysis was based on eighteen items that are believed to affect food security in the districts of Tororo and Busia. Coefficients were estimated for difficulty levels and also for the easiness parameters and also tested for significance as shown in Tables 3 and 4, respectively. Seven items of food security measurements used in this study were not significant for both districts.

The easiness parameter level estimates the beta coefficients to show the ease of accessing food in the districts. Similarly, at a five percentage level of significance, seven out of eighteen items were not significant. Although they were not significant, we did not have sufficient evidence to eliminate them from the analysis.

Table 5 shows descriptive analysis of the situation of food security in two districts. All households sampled in the study were carefully binomially classified. They either had a negative or a positive Rasch model estimated value. Households with positive Rasch model estimated values were classified as food secure while those with negative Rasch model values were classified as food insecure households. To obtain the overall cluster value, descriptive statistics were generated and comparisons were made as shown in Table 5. The analysis indicated that Tororo district average food security assessment is 0.137 ± 0.181 and Busia district is 0.768 ± 0.177 measured at a five percentage level of significance. Households in Busia district show a higher level of food security compared to those in Tororo district. Based on the size of the standard errors, the confidence interval for Tororo district stretches from negative to positive values. This implies that, on average, food insecurity in households found in Tororo district is higher than those households in Busia districts.

**Figure 1:**
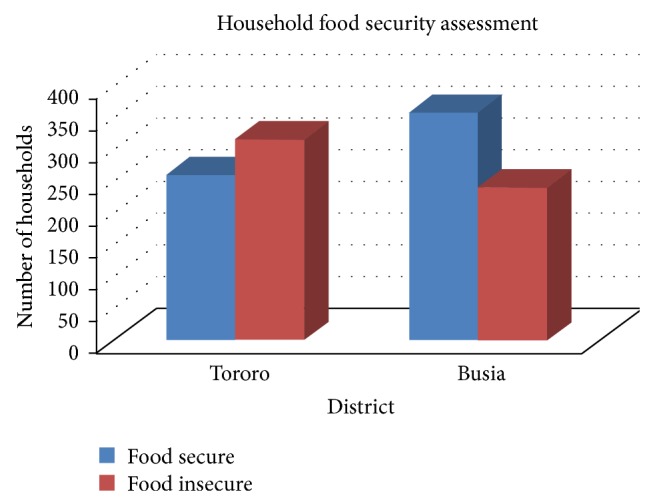
Household food security assessment for Tororo and Busia districts.

Household (individual) parameter estimates were generated to represent scores for food security measurements in households. The household food security values ranged between −4.933 to 4.097 and −3.919 to 4.225 with mean food security scores of 0.137 and 0.768 for districts of Tororo and Busia, respectively. Consequently, a binary household food security classification was developed whereby negative scores were categorised as food insecurity while nonnegative scores represented food secure households.  Figure 1 reveals that households in Busia district are relatively more food secure compared to those in Tororo district. The findings were in line with other surveys carried out by the Uganda Bureau of Statistics and supported by the fact that Busia district is a border district whose food security is determined by not only practising subsistence food production but also more involvement in general commercial activities. These findings further revealed that although more households in Tororo district were involved in food cultivation, it is not a sufficient condition for a household to be food secure.

**Figure 2:**
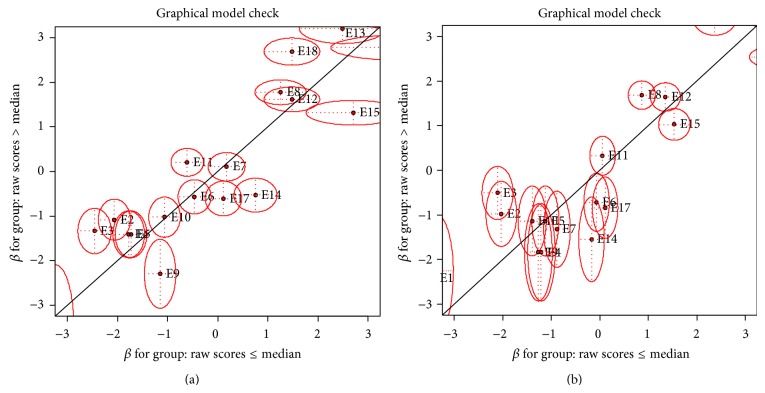
Graphical Rasch model check for Tororo (a) and Busia (b) districts.

Figure 2 shows a graphical model check aimed at establishing if the sample data for the two districts adequately fitted the Rasch model so as to be able to interpret the results. This graphical approach tests for subgroup homogeneity between two betas where raw scores are greater than the medium score against one where the raw scores are less or equal to the medium score. In both cases, there was no sufficient evidence to support subgroup heterogeneity; hence we concluded that the two subgroups in both districts were homogenous. This confirmed that there was a noticeable pattern of the way households responded to the different items, thus acting as consistent measures of food security.

Outfit is a chi-square statistic, divided by its degrees of freedom to have a mean-square form for ease of interpretation. Since the data is not heavily contaminated with irrelevant outliers, outfit statistics were used as a measure to determine the fit of the data to the Rasch model.  Table 6 shows that the average mean squares for districts of Tororo (0.932) and Busia (0.949) do not vary significantly from 1 and neither do they vary significantly between districts. Since all the mean square fit statistics were in the range of 0.5 to 1.5, none of them showed any signs of distortion, degradation, or less productivity for measurement. This was an indicator that items used in the study were very productive for measurement of food security.

The item characteristic curves, ICC, illustrate plots of the probability that the items would be answered in affirmative against the ability levels to handle the food insecurity situation in a household. The item plots on the extreme right correspond to higher levels of difficulty in handling food insecurity while those on the extreme left show lower levels of difficulty in coping with food insecurity situations in the districts of Tororo and Busia. For example, in both districts, items 1 and 11 corresponded to lower levels of difficulty while items 5, 13, and 16 corresponded to higher levels of difficulty. Implying that households could easily respond to items 1 and 11 in regard to food insecurity measurement, but responses to items 5, 13, and 16 were not very easily obtained in the food security assessment.

## 4. Discussion and Conclusion

The Rasch modelling approach was applied in this paper as a confirmatory approach with the following assumptions: unidimensional trait of the ability parameter of households to secure food, local independence of the eighteen items, and the response of a household to an item followed by a mathematical item response function. Since household neither answered all items in affirmative nor failed to affirm all items, the eighteen items were all used as candidates to fit the Rasch model. The average outfit mean square values of 0.932 and 0.949 for Tororo and Busia districts, respectively, are in the expected range of 0.5 to 1.5, hence showing a high degree of productivity for measurement of food security.

**Figure 3:**
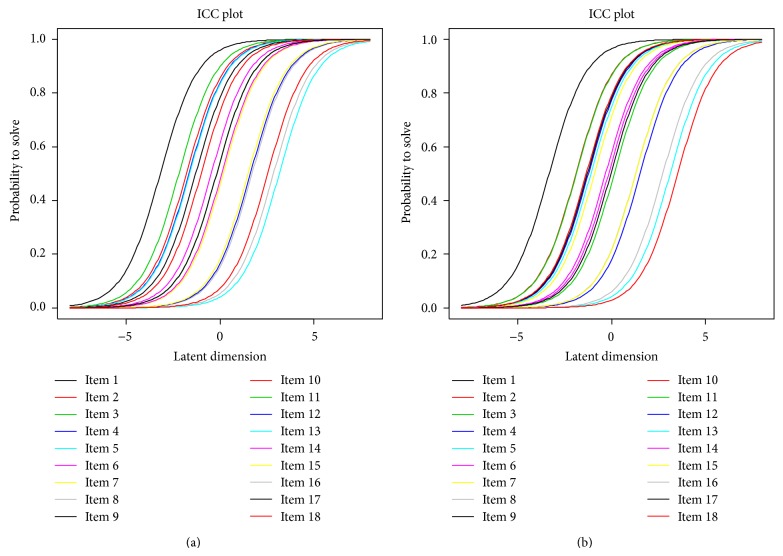
Item characteristic curves for Rasch model check for the districts of Tororo (a) and Busia (b).

All tests including the graphical model checks, the item characteristic curves, and the outfit mean squares confirm the suitability of the data in fitting the Rasch model to measure household food security in the two districts of Uganda. Further analysis could be done to compare the results of the Rasch measurement approach with other item analysis paradigms of item response theory and the classical test theory. From the item characteristic curves in Figure 3, it is evident that item ordering is important in measurement of household food security, thus increasing the difficulty of an item causing the curve to shift right. Households need to be more able to have the same chance of food security status. Being more able implies that households should be empowered with other means of income that will subsequently increase their ability towards being food secure.

Generalized mixed models (GLMM) have been used to develop food insecurity scales for measurement of food insecurity in three regions of Bangladesh and compared it with the Rasch model [27]. The GLMM included demographic variables and income as covariates and found all of them, except CHILD, significant implying that the demographic variables increased the likelihood of food insecurity. The interactions though statistically significant did not lead to much change in the results when interactions were excluded. GLMM and the Rasch model correlations were high (0.9976) showing that the additional variables used in the GLMM did not result in much change in food insecurity when compared with the results obtained using the Rasch model. While the GLMM was able to quantify the effect of household characteristics on food insecurity, Rasch model was able to predict food insecurity level for specific households. GLMM could not be used to estimate proportion of a population corresponding to a particular food security level.

Household-level method involving use of small area estimation technique in multivariate regression models documented by [28–31] is also used in food insecurity assessments. It requires a minimum of two sets of data: household-level census data and a representative household survey corresponding approximately to the same period as the census. The first step was to estimate a model of consumption-based household welfare using household survey data with explanatory variables limited to those found in both datasets. The resulting parameter estimates were applied to the census data. For each household, the estimated parameters from the regression were used to compute the probability of each household in the census living in poverty. The household-level value of the explanatory variable was multiplied by the corresponding parameter estimate. The estimated value of the benchmark indicator was then used to determine the probability of a household being food insecure or poor in terms of a given threshold below which a household was food insecure.

The challenge with this method is that it requires two sets of data which should have been collected during the same periods of time. Getting variables which match in the two datasets could be a challenge unless they are planned together with the intention of using the two for the purpose of small area estimation. The Rasch model requires one dataset that can be more easily collected and analysed.

The authors analysed the same data using logit models with different proxies for food insecurity including food stored, access to food, food harvested, and a hybrid dummy variable that combined the three. The paper [20] discusses the logit model results.

The Rasch model results (Figure 1) show that slightly more households in Tororo district are food insecure while in Busia, there are more food secure households than food insecure households. This is a significant contrast with the logit model classification which showed that there were many more households that were food insecure in both districts [20]. This contrast in results could be due to the differences in what the models measure. The Rasch model variables are about attitudes, feelings, and perceptions on the food security situation, which are intangible and quite subjective. On the other hand, the logit model combines these variables used in the Rasch model with variables which assess household demographic, social, and economic characteristics which are tangible. Such variables not included in the Rasch model are food production trends, frequency of eating meals, type of foods eaten, household income, sources of income total land area cultivated, and possible loan/lending arrangements. The logit model is more robust in the sense that it includes more factors that influence the livelihood of a household although [27] while assessing differences between GLMM and Rasch model found out that these extra factors did not change the food security status much.

The advantage of the Rasch model is that it is able to estimate parameters even when there is nonresponse or when there are different but partially overlapping response items. In situations where the items have different discriminating power, Rasch model can easily generalize by assuming that each respondent randomly guesses the answers to some or all the items. Parameter separation property of the model means that item severity does not depend on the specific households used in the calibration. The limitation of the Rasch model is that it does not cover all potential household food insecurity experiences as it concentrates on perceptions, attitudes, and feelings. It does not allow gender, household or demographic characteristics, and their interactions as covariates in the model when computing individual household food security scores. The effects of such factors on food security are therefore not estimated. The logit model, like the GLMM models, has the advantage that it includes all the items of the Rasch model and also incorporates the demographic, socioeconomic, cultural, and related variables. For the Rasch model, if a respondent does not affirmatively answer the questions, their food insecurity status cannot be determined using the model so they have to be excluded. Rasch scale being a single one-dimensional scale leaves out from the analysis valuable household food insecurity experiences that are multidimensional. An example is culture which takes on different meanings in different ethnic communities. The same perceptions or behaviours in one culture do not necessarily indicate the same degree of relative household food insecurity in another culture. As a result, generalization from the model could be misleading. The logit model like the GLMM models is not unidirectional and incorporates a wide range of variables representing different aspects of food security. The aspects of a factor that may not have been covered by one variable may be taken care of by another variable. An example is food availability variables. Value of food harvested, expenditure on food during the season in question, contributions of household members to the household food basket, land area cultivated, and land area available can all be incorporated in the model and are comprehensive enough to cover aspects of food availability. Unlike the classical Rasch model which uses categorical variables, the logit model can take on mixed (categorical and continuous) variables.

In this paper the analysis was based on a two-parameter level dichotomous Rasch model to assess food security. However, an extension to modelling ordered polytomous item responses rather than dichotomous item response functions could be considered. Furthermore, consideration would be given to fit models that allow for different latent distributions for households with children and those without children and possibly other subgroups of respondents. This would highlight the importance of family size in influencing household's food security in the geographical scope of the study.

## Figures and Tables

**Table 1:** Study variables and descriptions.

Variable	Variable description
E1.	We worried whether our food would run out before we got money to buy more.

E2.	The food that we harvested or bought just didn't last, and we didn't have money to get more.

E3.	We couldn't afford to eat balanced meals.

E4.	We relied on only a few kinds of low-cost food to feed our child/children because we were running out of food and money to buy food.

E5.	We couldn't feed our child/the children a balanced meal, because we couldn't afford that.

E6.	Our child was/children were not eating enough because we just couldn't afford enough food.

E7	Did you/or [*sic*] other adults in your household ever cut the size of your meals or skip meals because there wasn't enough food or money for food?

E8.	If Yes to E7, how often did this happen?

E9.	Did you ever eat less than you felt you should because there wasn't enough money to buy food?

E10.	Were you every [*sic*] hungry but didn't eat because you couldn't afford enough food?

E11.	Did you lose weight because you didn't have enough money for food?

E12.	Did (you/you [*sic*] or other adults in your household) ever not eat for a whole day because there wasn't enough food or money for food?

E13.	If Yes to E12, how often?

E14	Did you ever cut the size of (your child's/any of the children's) meals because there wasn't enough food or money for food?

E15.	Did any of the children ever skip meals because there wasn't enough food or money for food?

E16.	If yes to E15, how often did it happen?

E17.	Was your child/were the children ever hungry but you just couldn't afford more food?

E18.	Did your child/any of the children ever not eat for a whole day because there wasn't enough money for food?

**Table 2:** The data structure.

Respondent	E1	E2	E3	E4	E5	E6	E7	E8	⋯	⋯	⋯	E18
1	1	1	1	1	1	1	0	0				0
2	1	1	1	0	1	1	1	1				1
3	1	1	0	0	0	0	1	1				0
4	1	0	1	1	1	0	1	1				0
5	0	1	0	1	1	0	0	0				0
6	1	1	1	1	1	1	1	0				1
7	1	1	1	1	1	1	1	1				1
8	1	1	1	1	1	1	1	1				1
9	1	1	0	1	0	0	0	0				0
10	1	1	1	1	1	1	1	1				1
⋮	⋮	⋮	⋮	⋮	⋮	⋮	⋮	⋮				⋮
⋮	⋮	⋮	⋮	⋮	⋮	⋮	⋮	⋮				⋮
⋮	⋮	⋮	⋮	⋮	⋮	⋮	⋮	⋮				⋮
1175	1	1	1	1	1	1	1	1				1

**Table 3:** Estimated theta coefficients of the Rasch model for Tororo and Busia districts.

Theta (difficulty parameter level) estimates						
Item	Estimate for Tororo	SE		Estimate for Busia	SE	
E2	−1.793	0.134	∗∗	−1.9	0.15	∗∗
E3	−2.191	0.141	∗∗	−1.877	0.149	∗∗
E4	−1.651	0.131		−1.237	0.134	
E5	−1.616	0.131		−1.108	0.131	
E6	−0.471	0.119		−0.166	0.117	
E7	0.164	0.116		−0.916	0.128	
E8	1.701	0.123	∗∗	1.271	0.11	∗∗
E9	−1.297	0.126		−1.293	0.135	
E10	−1.011	0.123		−1.351	0.136	
E11	−0.185	0.117	∗∗	0.133	0.114	
E12	1.612	0.122		1.51	0.111	
E13	3.15	0.156		3.11	0.133	∗∗
E14	0.081	0.116	∗∗	−0.323	0.119	∗∗
E15	1.496	0.12	∗∗	1.282	0.11	∗
E16	2.842	0.146		2.723	0.125	∗∗
E17	−0.185	0.117	∗∗	−0.041	0.115	∗
E18	2.531	0.137	∗∗	3.51	0.144	

Source: primary data from the survey.

^*^implies 0.05 and ^**^implies 0.01 level of significance.

**Table 4:** Estimated beta coefficients of the Rasch model for Tororo and Busia districts.

Beta (easiness parameter level) estimates						
Item	Estimate for Tororo	SE	Sign.	Estimate for Busia	SE	Sign.
E1	3.177	0.169	∗∗	3.328	0.197	∗∗
E2	1.793	0.134	∗∗	1.900	0.150	∗∗
E3	2.191	0.141	∗∗	1.877	0.149	∗∗
E4	1.651	0.131		1.237	0.134	
E5	1.616	0.131		1.108	0.131	
E6	0.471	0.119		0.166	0.117	
E7	−0.164	0.116		0.916	0.128	
E8	−1.701	0.123	∗∗	−1.271	0.110	∗∗
E9	1.297	0.126		1.293	0.135	
E10	1.011	0.123		1.351	0.136	
E11	0.185	0.117	∗∗	−0.133	0.114	
E12	−1.612	0.122		−1.510	0.111	
E13	−3.150	0.156		−3.110	0.133	∗∗
E14	−0.081	0.116	∗∗	0.323	0.119	∗∗
E15	−1.496	0.120	∗∗	−1.282	0.110	∗
E16	−2.842	0.146		−2.723	0.125	∗∗
E17	0.185	0.117	∗∗	0.041	0.115	∗
E18	−2.531	0.137	∗∗	−3.510	0.144	

Source: primary data from the survey.

^*^implies 0.05 and ^**^implies 0.01 level of significance.

**Table 5:** Descriptive statistics for household food security for Tororo and Busia districts.

Household score on food security scale		
Descriptive statistics	Tororo	Busia
Mean	0.137	0.768
Standard error	0.092	0.090
Median	0.290	1.027
Mode	2.008	2.558
Standard deviation	2.040	2.078
Sample variance	4.163	4.317
Kurtosis	−0.663	−0.315
Skewness	−0.190	−0.419
Range	8.030	8.144
Minimum	−3.933	−3.919
Maximum	4.097	4.225
Largest (10)	4.097	4.225
Smallest (10)	−3.933	−3.919
Confidence level (95%)	0.181	0.177

Source: primary data from the survey.

**Table 6:** Rasch model item fit statistics for Tororo and Busia districts.

Item	Tororo district (df = 489)		Busia district (df = 530)	
	Outfit	MSQ	Outfit	MSQ
E1	0.803	1.116	2.214	1.222
E2	3.876	1.035	1.088	0.989
E3	3.981	1.121	3.286	1.293
E4	1.749	0.952	0.672	0.882
E5	1.124	1.027	0.805	1.114
E6	0.782	0.903	0.784	0.71
E7	0.777	0.889	0.616	0.845
E8	1.181	1.093	1.175	1.182
E9	0.547	0.685	0.569	0.738
E10	0.853	0.86	0.679	0.809
E11	1.487	1.244	1.386	1.181
E12	0.82	0.886	1.075	1.055
E13	1.168	0.823	2.017	1.015
E14	0.521	0.686	0.607	0.722
E15	0.68	0.732	0.624	0.829
E16	0.576	0.817	0.496	0.722
E17	0.803	0.78	0.754	0.759
E18	2.313	1.133	0.912	1.015

Average score	1.336	0.932	1.098	0.949

Source: primary data from the survey.
